# Dichlorvos exposure impedes extraction and amplification of DNA from insects in museum collections

**DOI:** 10.1186/1742-9994-7-2

**Published:** 2010-01-18

**Authors:** Marianne Espeland, Martin Irestedt, Kjell Arne Johanson, Monika Åkerlund, Jan-Erik Bergh, Mari Källersjö

**Affiliations:** 1 Swedish Museum of Natural History, Entomology Department, Box 50007, SE-104 05 Stockholm, Sweden; 2 Stockholm University, Zoological Institute, SE-106 09 Stockholm, Sweden; 3 Swedish Museum of Natural History, Molecular Systematics Laboratory, Box 50007, SE-104 05 Stockholm, Sweden; 4 Swedish Museum of Natural History, Research Department, PRE-MAL, Box 50007, SE-104 05, Stockholm, Sweden; 5 Dalarna University College, SE-791 88 Falun, Sweden; 6 Current address: Göteborg Botanical Garden, Carl Skottsbergs Gata 22 A, SE-413 19 Gothenburg, Sweden

## Abstract

**Background:**

The insecticides dichlorvos, paradichlorobenzene and naphthalene have been commonly used to eradicate pest insects from natural history collections. However, it is not known how these chemicals affect the DNA of the specimens in the collections. We thus tested the effect of dichlorvos, paradichlorobenzene and naphthalene on DNA of insects (*Musca domestica*) by extracting and amplifying DNA from specimens exposed to insecticides in two different concentrations over increasing time intervals.

**Results:**

The results clearly show that dichlorvos impedes both extraction and amplification of mitochondrial and nuclear DNA after relatively short time, whereas paradichlorobenzene and naphthalene do not.

**Conclusion:**

Collections treated with paradichlorobenzene and naphthalene, are better preserved concerning DNA, than those treated with dichlorvos. Non toxic pest control methods should, however, be preferred due to physical damage of specimens and putative health risks by chemicals.

## Background

Natural history collections are an invaluable source of biological data [1-3]. These collections record the distribution of known taxa in space and time and document both what we know and what we don't know about the world's biota [4]. Biologists all over the world have been extracting ecological, morphological, phylogenetic, diversity and biogeographic data from museum specimens for decades, if not decennia [1]. More recently these specimens are also in frequent use for the extraction of DNA in e.g. molecular phylogenetic, population genetic and conservation genetic studies [5-9]. It could also be expected that Natural history collections will be much more important in molecular studies in the near future owing to; 1) difficulties to collect fresh biological material from many regions and the extinction of taxa due to habitat loss, and 2) the development of new high-throughput sequencing methods [10] and protocols that makes it possible to use these techniques for PCR-product sequencing [11] and conducting extensive molecular studies based on fragmented DNA in museum collections.

Museum collections are prone to attacks by insect pests, especially beetles of the family Dermestidae (Coleoptera). If left unattended these pests can completely destroy an insect collection within a few months time. Hence a variety of methods have been developed to eradicate the pest insects e.g. fumigation or other treatments with insecticides [12,13], traps [14-16], heating [17-19] or freezing of infested specimens [20-22] and modified atmosphere [23-28].

Many different insecticides have been used in eradication of pest insects in collections. The use is declining, but it is still utilized in many museums [29,30]. Several studies of the effects of insecticides on the pest insects e.g. [12,31] and their effect on different materials in museum collections [32,33] have been performed, but there are few studies of how insecticides affect the DNA of the specimens in natural history collections. Whitten et al [34] found no effect of sulphuryl fluoride (Vikane) on the DNA of herbarium specimens. According to Kigawa et al. [35] methyl bromide, ethylene oxide, propylene oxide and methyl iodide all affected the DNA in both freeze-dried mushrooms and chicken muscle negatively, whereas sulphuryl fluoride did not. To our knowledge no studies on the effects of insect DNA have been performed.

Naphthalene, paradichlorobenzene and dichlorvos are some of the most frequently used insecticides in insect collections, but their effect on the DNA of insect specimens is not known. We therefore exposed dried insects to various concentrations of these insecticides over a period of 20 months (605 days), extracted DNA from the specimens and ran both total DNA extracts and polymerase chain reaction (PCR) products on agarose gels to investigate effects of these insecticides on the DNA of insect specimens.

## Methods

**Figure 1:**
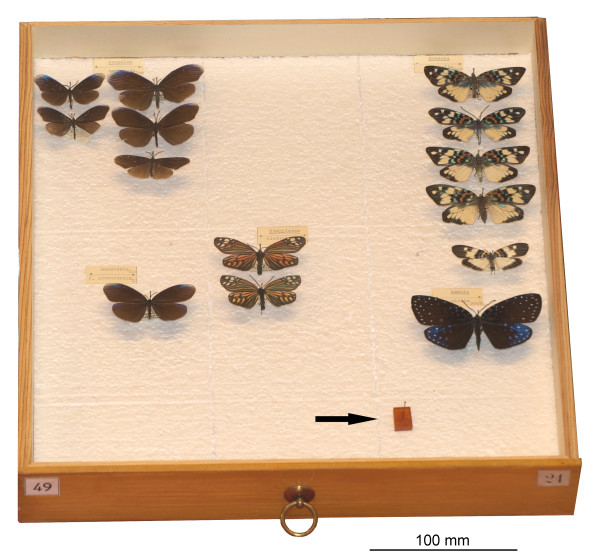
**Dichlorvos (arrow) as used in insect drawers at the Swedish Museum of Natural History**.

Common houseflies (*Musca domestica*) were dried on silica gel for three weeks and then exposed to one of eight different treatments (Table 1). Insecticides were placed in 15 cm^3 ^glass vials under a piece of cotton. Flies were placed on the cotton to avoid direct exposure to the insecticide. Vials where then sealed with plastic lids with silicone insulation to make them air tight and stored at room temperature. Recommended dosage and 10× recommended dosage of insecticides were calculated based on information on the insecticide containers. Recommended dosage for naphthalene and paradichlorobenzene were 150 g/m^3 ^air and 1.6 g/m^3 ^for dichlorvos. We used 15 cm^3 ^vials in the experiments so these amounts transferred to 0.002 g/vial for naphthalene and paradichlorbenzene and 2.4*10^-4^g/vial for dichlorvos. We did not have accurate enough equipment to measure as small amounts as the latter thus we used 0.001 g/vial which corresponds to roughly 41× the recommended dosage of dichlorvos. This might seem like a very high quantity, but it is justified since much higher doses of dichlorvos are used in real collections. A standard insect drawer in use at the Swedish Museum of Natural History has a volume of 6800 cm^3 ^(6.8 l). This means that recommended dosage of one drawer should be 1 g for naphthalene and dichlorvos and as little as 0.01 g for dichlorvos. Considerably higher doses have been used in drawers at the Swedish Museum of Natural History (Figure 1). The potency of dichlorvos makes it virtually impossible to dose it correctly.

**Table 1:** The six insecticide treatments and controls in the current study.

	I Dichlorvos	II Paradichlorbenzene	III Naphthalene	IV Control
1 High concentration	0.02 g/vial	0.02 g/vial	0.02 g/vial	NA
2 Low concentration	0.001 g/vial	0.002 g/vial	0.002 g/vial	NA

In addition to recommended dosage we also included a treatment with 10× (833× for dichlorvos) recommended dosage (0.02 g/vial) and controls without insecticides. Samples were taken with increasing intervals over a time period of 20 months (605 days) and DNA extracted according to the scheme in Table 2.

**Table 2:** Extraction dates and length of pesticide exposure (in days) for all samples.

Sample	Extraction date	Pesticide exposure (days)
1	**17/04/07**	**1**
2	18.4-2007	2
3	19.4-2007	3
4	20.4-2007	4
5	22.4-2007	6
6	24.4-2007	8
7	**26.4-2007**	**10**
8	30.4-2007	14
9	8.5-2007	22
10	**27.5-2007**	**41**
11	11.7-2007	86
12	**28.8-2007**	**134**
13	14.10-2007	181
14	**1.12-2007**	**229**
15	**18.1-2008**	**278**
16	**6.3-2008**	**326**
17	**23.4-2008**	**374**
18	**10.6-2008**	**422**
19	**10.12-2008**	**605**

Samples shown on gels in this paper are given in bold.

### Molecular procedures

DNA was extracted from whole houseflies using the Qiagen DNeasy Tissue Extraction kit (Qiagen Inc., Valencia, California) which yields DNA fragments of length 50 000 kb and shorter. Twelve μl of the aliquots were run directly on 1% agarose gels in 0.5× TBE buffer for 5 hours and visualized under UV light.

Fragments of comparable length of one mitochondrial (COI, 658 bp; primers LCO-HCO [36]) and one nuclear gene (EF1a, 716 bp; primers M46.1-R [37,38]) were amplified using Ready-To-Go™ PCR Beads (Amersham Pharmacia Biotech, Piscataway, New Jersey). Reaction mixtures consisting of 2 μl template, 1 μl primer (10 μm, forward and reverse) 16 μl dH_2_0 and beads were heated to 95°C for 5 minutes, followed by 40 cycles of 30 seconds at 95°C, 30 seconds at a specific annealing temperature (52°C for EF1a and to 50°C for COI) and 50 seconds at 72°C, and then a final extension of 8 minutes at 72°. PCR products were visualized by ultraviolet light on a 0.8% agarose gel after electrophoresis.

If fragmentation is seen in both extraction and amplification then there is evidence that these insecticides cause degradation of DNA. If, on the other hand, initial gel runs on extracts exposed to insecticides are identical to controls, but amplification of genes are impossible or very difficult we have evidence that insecticides might inhibit amplification.

## Results

### Effect on total DNA

**Figure 2:**
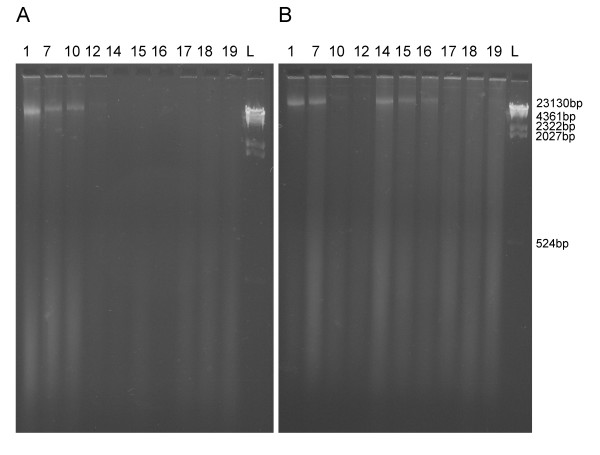
**Total DNA extracts of dichlorvos exposed specimens**. A) High concentration (0.02 g/vial). B) Low concentration (0.001 g/vial). L indicates ladder. See Table 2 for sample intervals.

**Figure 3:**
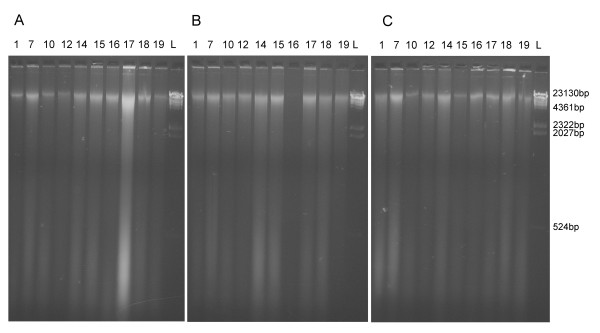
**Total DNA extracts of specimens exposed to high concentration (0.02 g/vial) A) paradichlorobenzene and B) naphthalene, and C) controls not exposed to insecticides**. L indicates ladder. See Table 2 for sample intervals.

Visualization of DNA extracts on agarose gels showed that dichlorvos fragments DNA both in high and low concentration (Figure 2A-B). After four and twelve months of exposure of the high and recommended dosage dichlorvos respectively, the band of DNA of length around 23 000 bp, which constitutes of most of the DNA in the control, has completely disappeared from the dichlorvos samples. Only a very low amount of highly degraded DNA (<500 bp) is present in these samples. No effect on DNA was seen in samples treated with naphthalene and paradichlorobenzene (Figure 3A, B, only high concentration, 0.02 g/vial, shown; control: Figure 3C).

### Amplification of nuclear and mitochondrial DNA

**Figure 4:**
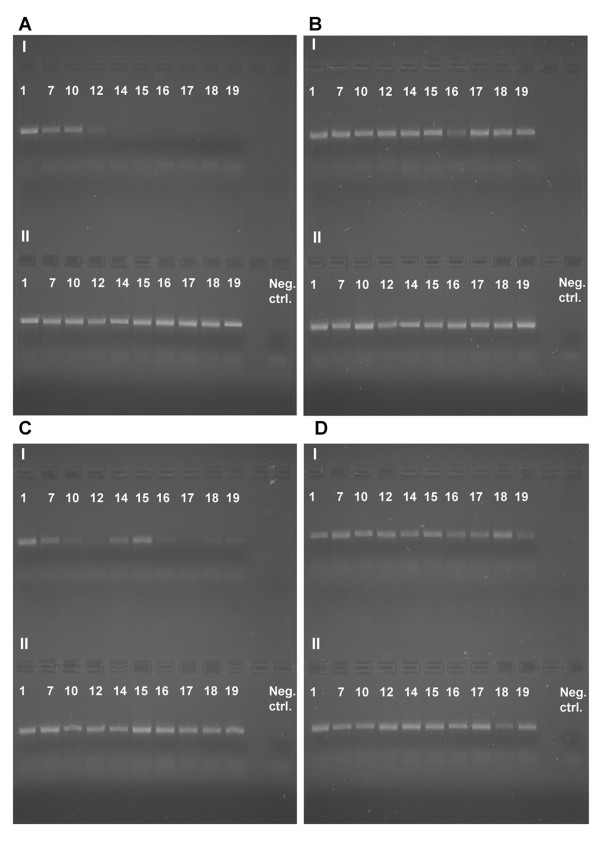
**Amplification of a 717 bp fragment of the nuclear gene EF1a**. A-I) High concentration dichlorvos, A-II) High concentration paradichlorobenzene, B-I) High concentration naphthalene, B-II) Control, C-I) Low concentration dichlorvos, C-II) Low concentration paradichlorobenzene, D-I) low concentration naphthalene, D-II) Control. See Table 2 for sample intervals.

**Figure 5:**
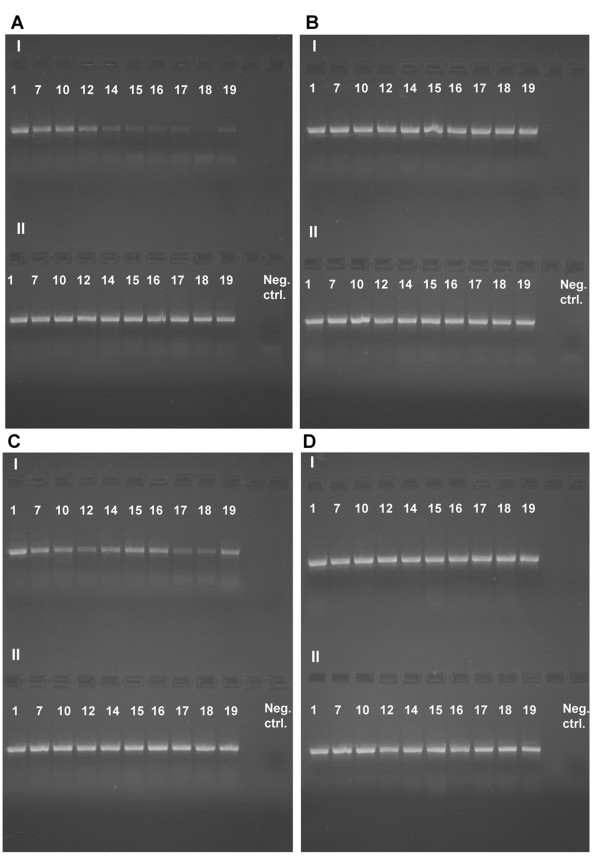
**Amplification of a 658 bp fragment of the mitochondrial gene COI**. A-I) High concentration dichlorvos, A-II) High concentration paradichlorobenzene, B-I) High concentration naphthalene, B-II) Control, C-I) Low concentration dichlorvos, C-II) Low concentration paradichlorobenzene, D-I) low concentration naphthalene, D-II) Control. See Table 2 for sample intervals.

After 134 days (sample 12, Figure 4A-I) of dichlorvos exposure (high concentration) amplification of EF1a is considerably impeded and after 229 days (sample 14, Figure 4A-I) it is no longer possible. Amplification of COI is impeded after 229 days (sample 14, Figure 5A-I) of dichlorvos exposure (high concentration). Very weak bands are, however, visible during the whole experiment (605 days) so amplification is possible, but made more difficult. When looking at the samples exposed to lower concentration of dichlorvos the results are less conclusive but amplification of both EF1a (Figure 4C-I) and COI (Figure 5C-I) is impeded by dichlorvos even here, indicated by weaker bands, especially for EF1a, for samples treated with dichlorvos than for the controls (Figures 4B-II, 4D-II). When compared with the controls (EF1a: Figure 4B-II, 4D-II; COI: Figure 5B-II, 5D-II), naphtalene (EF1a: Figures 4B-I, 4D-1; COI: Figures 5B-I, 5D-1) and paradichlorobenzene (EF1a: Figures 4A-II, 4C-II; COI: Figures 5A-II, 5C-II) do not seem to affect the amplification of neither EF1a nor COI.

## Discussion

The use of DNA from organisms in museum collection is increasing and it is thus important to curate the collections with this in mind. Dichlorvos clearly affects the DNA of insects negatively already after four months of exposure and the effect increases over time, whereas naphthalene and paradichlorobenzene do not seem to affect DNA, at least not over a time period of 20 months. Negative effects on DNA are observed both in total DNA extractions and amplification of nuclear and mitochondrial DNA, thus the major problem is fragmentation of DNA and not inhibition of PCR primers. Effects are also larger for the nuclear gene than for the mitochondrial gene, which is not unlikely since the mitochondrial gene is present as multiple copies in every cell, whereas nuclear DNA only in two copies. Mitochondria are also structurally strong which might lead to better preservation of mitochondrial DNA than its nuclear counterpart [39]. The concentration of insecticide used is also important with higher concentration resulting in increased damage of DNA. The dosages of dichlorvos used in this study might seem extremely high, but they (even the high dose) are probably closer to reality than the recommended dose. The pesticide is very potent even in small doses, and it is almost impossible not to use more than necessary. It is also possible that we will see similar results of DNA fragmentation for paradichlorobenzene and naphthalene when used in higher doses. Dichlorvos is a potent acetylcholinesterase inhibitor and can cause DNA damage in human cells at low concentrations, even after short exposure [40,41], and it is putatively carcinogenic in humans [42]. It has also been shown to cause severe damage on museum material, such as bleaching of colour, and even corrosion of metal [32,33]. Because of its deleterious effects to both human and insect DNA the use of dichlorvos for pest prevention in natural history collections should be strongly avoided. Even naphthalene and paradichlorbenzene, are suspected carcinogens [43,44]. They also effect colours and soften resins [45], and are documented less effective in killing the pests than dichlorvos [31]. Therefore they are not recommended for use in museums. Non-toxic methods such as freezing [21,22], or anoxic treatment [27] should be recommended if infestation has occurred since they are effective against pests and at the same time little hazardous to humans and items. On the other hand we wholeheartedly agree with Blyth & Smith [46], that prevention is better than the cure.

## Conclusion

The use of dichlorvos for pest eradication in natural history collections should be strongly avoided due to deleterious effects on DNA. Chemical eradication methods in general should be avoided since they can cause damage to specimens and are associated with putative health issues.

## Competing interests

The authors declare that they have no competing interests.

## Authors' contributions

MI, KJE, MÅ, J-EB and MK conceived the project. ME set up the experiment, did the molecular work and wrote the paper. MI, ME and KJE discussed the molecular work. All authors discussed the experimental setup and read and approved the final manuscript.
